# HINT1 in Neuropsychiatric Diseases: A Potential Neuroplastic Mediator

**DOI:** 10.1155/2017/5181925

**Published:** 2017-10-30

**Authors:** Peng Liu, Zhongwei Liu, Jiabei Wang, Xiancang Ma, Yonghui Dang

**Affiliations:** ^1^College of Medicine & Forensics, Key Laboratory of the Health Ministry for Forensic Medicine, Key Laboratory of Environment and Genes Related to Diseases of the Education Ministry, Xi'an Jiaotong University Health Science Center, Xi'an 710061, China; ^2^Department of Cardiology, Shaanxi Provincial People's Hospital, Xi'an 710068, China; ^3^Department of Pharmaceutical Sciences, School of Pharmacy, University of Maryland Baltimore, Baltimore MD 21201, USA; ^4^Department of Psychiatry, First Affiliated Hospital of Xi'an Jiaotong University Health Science Center, Xi'an, China

## Abstract

Although many studies have investigated the functions of histidine triad nucleotide-binding protein 1 (HINT1), its roles in neurobiological processes remain to be fully elucidated. As a member of the histidine triad (HIT) enzyme superfamily, HINT1 is distributed in almost every organ and has both enzymatic and nonenzymatic activity. Accumulating clinical and preclinical evidence suggests that HINT1 may play an important role as a neuroplastic mediator in neuropsychiatric diseases, such as schizophrenia, inherited peripheral neuropathies, mood disorders, and drug addiction. Though our knowledge of HINT1 is limited, it is believed that further research on the neuropathological functions of HINT1 would eventually benefit patients with neuropsychiatric and even psychosomatic diseases.

## 1. Introduction to HINT1

Proteins containing the histidine triad (HIT) motif, a conserved HisXHisXHis sequence (in which X represents any hydrophobic amino acid), constitute an enzyme superfamily known as the HIT proteins [1]. According to enzyme activity classification, HIT proteins can be classified into three branches: nucleoside phosphoramidate hydrolases, dinucleotide hydrolases, and nucleotidylyl transferases. HIT proteins are conserved throughout evolution, and more than 35 members of this superfamily have been found in 29 species, including bacteria, archaea, yeast, plants, *C. elegans*, *Drosophila*, and mammals, implying that HINT1 exerts basic and essential physiological functions [2]. The human genome encodes seven HIT proteins, which mainly serve as nucleoside transferases and hydrolases and can be divided into five classes: histidine triad nucleotide-binding protein (HINT), galactosyl-1-phosphate uridine acyltransferase, aprataxin, DCPS/DCS-1, and the brittle histidine triad proteins [3–6].

**Figure 1:**
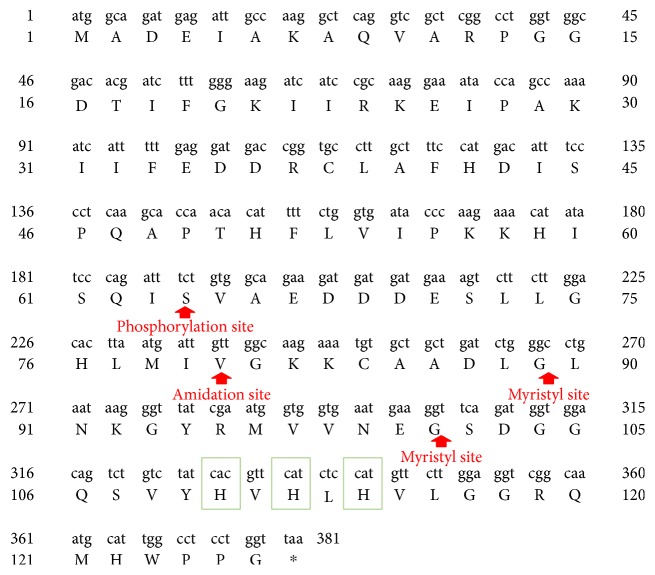
DNA and protein sequences of human HINT1. Green boxes indicate the histidine triad (HIT) domain. The potential modification sites of the protein are shown, including the phosphorylation site, amidation site, and myristyl site.

**Figure 2:**
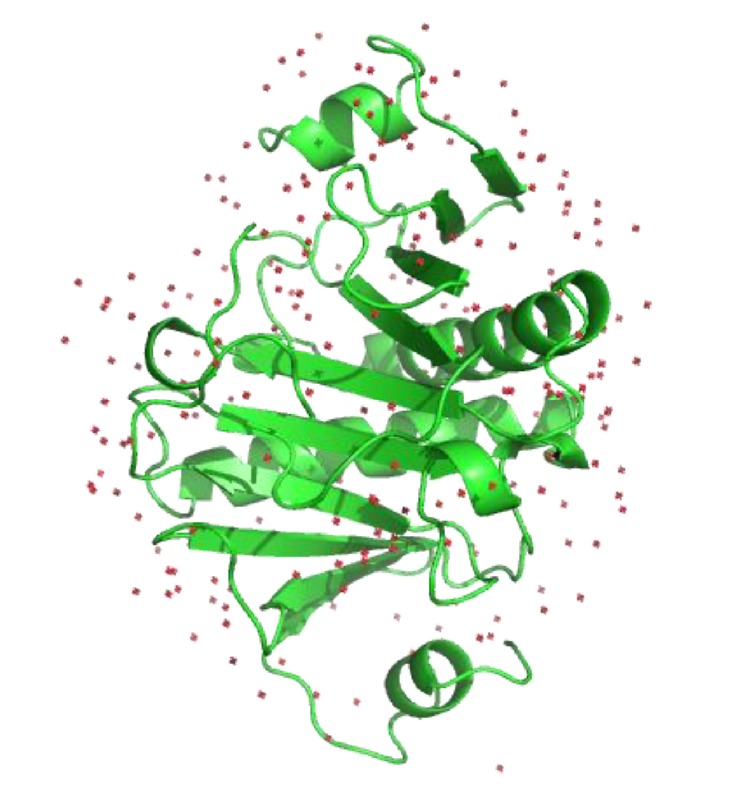
Crystal diffraction pattern of the HINT1 protein. HINT1 consists of a homodimer, each subunit of which contains two *α*-helices and three *β*-sheets. From http://www.rcsb.org/pdb.

The histidine triad nucleotide-binding protein (HINT) (including human HINT and nonhuman Hint) is the first class of HIT superfamily. It is now suggested that at least one *HINT* gene is thought to exist in all sequenced genomes. Three independent HINT genes encoding the HINT1, HINT2, and HINT3 proteins are found in the human genome. Genes encoding HINT1 proteins are localized on human chromosome 5q31.2, with a full length of 6160 bp, containing three exons. HINT1 mRNA is composed of 782 bp, encoding a 126-amino acid cytosolic protein molecule with a relative molecular mass of approximately 14 kDa (Figure 1) [3, 7]. According to nuclear magnetic resonance (NMR) and crystallography studies, HINT1 is one of the purine nucleotide-binding proteins. Two subunits constitute a homodimer structure with a binding site for purine bases and a binding site for ribose on each subunit (Figure 2) [1, 8, 9].

HINT1 was first described as a protein kinase inhibitor in 1990 [10] and supposed to be protein kinase C inhibitor 1 (PKCI-1) in early literature [11, 12]. Although direct or indirect interactions between HINT1 and protein kinase C (PKC) were suggested, the inhibitory effects of HINT1 on PKC are still doubtful. Therefore, PKCI-1 was renamed to HINT1 [1]. The abbreviation PKCI is still occasionally used in the current literature with the altered meaning of protein kinase C interacting protein 1, which was indicated in Klein et al.'s study [13].

## 2. HINT1 in Tumorigenesis

Although HINT1 is known for its enzyme activity, studies have shown that it has additional functions unrelated to its enzymatic activity [14], such as tumorigenesis suppression [15, 16]. Accumulating evidence indicates that HINT1 plays a role as a haploinsufficient tumor suppressor in multiple malignant diseases, but little is known about the mechanism [15, 16]. Several recent studies demonstrated that by inhibiting the activities of transcription factors including AP1 [17], TFIIH [18], MITF [19], and USF2 [20], HINT1 could regulate gene expression in the Wnt/beta-catenin signaling pathway [21]. Therefore, it seems that HINT1 may exert potential anticancer effects as a gene transcriptional regulator. Currently, studies are focusing on the clinical relevance of HINT1 expression in several human specific cancers [22–24].

## 3. HINT1 in Neuropsychiatric Disorders

HINT1 has a wide range of distribution in various tissues including liver, kidney, stomach, and brain in humans and rodents [13]. HINT1 is expressed in the central nervous system (CNS) of mice and is particularly enriched in the olfactory sensory system, cerebral cortex, hippocampus, part of the thalamus, hypothalamus, midbrain, pons, and medulla oblongata [25]. The distribution of HINT1 in the CNS provides anatomical evidence for its potential importance in neuronal function.

### 3.1. Schizophrenia

The HINT1 gene is located on a genetic locus highly associated with schizophrenia (5q31.2) [26, 27]. Schizophrenia is a common psychiatric disease with manifestations of positive symptoms (hallucinations, delusions, disorganized speech, disorganized behavior, catatonic behavior, agitation, etc.) and negative symptoms (blunted affect, emotional withdrawal, apathetic social withdrawal, stereotyped thinking, attentional impairment, etc.), as well as cognitive, affective, and aggressive symptoms [28]. The etiology of schizophrenia is complicated, including epigenetic changes and interactions between genetic susceptibility and environment [29].

Vawter et al. found that HINT1 was significantly decreased in the dorsolateral prefrontal cortex (DLPFC) and prefrontal cortex in patients with schizophrenia [30–32]. Notably, the HINT1 gene is located in the SPEC2/PDZ-GEF2/ACSL6 region of 5q22-23, which is associated with schizophrenia [33]. The same team then evaluated eight single nucleotide polymorphisms (SNPs) in the HINT1 gene in Irish study of high-density schizophrenia families (ISHDSF, 1350 subjects and 273 pedigrees) and Irish case-control study of schizophrenia (ICCSS, 655 patients and 626 controls). They further compared expression levels of HINT1 in postmortem brain samples provided by the Stanley Medical Research Institute and concluded that mutations in the HINT1 gene were potentially correlated with schizophrenia [7]. Varadarajulu et al. [34] found that the expression of HINT1 protein was upregulated in the thalamus but downregulated in the DLPFC in postmortem brain samples of patients with schizophrenia compared to those of healthy controls, consistent with results from another study in 2011 [35]. Additionally, findings from the abovementioned studies suggest that the association between HINT1 and schizophrenia is gender-specific and may only exist in male patients [7, 32, 33].

The results obtained from clinical studies are further supported by studies of HINT1 knockout (KO) mice. Barbier and colleagues [36] demonstrated that compared with wild-type (WT) mice, HINT1 KO mice were more sensitive to acute amphetamine- (AMPH-) induced hyperlocomotor behavior. Quantitative microdialysis of the kinetics of dopamine (DA) in the striatum or nucleus accumbens (NAc) showed that presynaptic DA neurotransmission in these regions did not underlie the AMPH-induced behavioral phenotype of KO mice. However, systemic administration of apomorphine, a dopamine receptor agonist, significantly increased KO mouse locomotor activity, suggesting that the postsynaptic DA transmission may be dysregulated in KO mice. Considering that schizophrenia is often accompanied by dopaminergic system hyperfunction [37] and the hyperactivity induced by AMPH represents the positive symptom-like behavior in rodent models for schizophrenia [38], HINT1 KO mice appear to be a useful genetic animal model for studying schizophrenia. Furthermore, we found that HINT1 plays a role in a social isolation (SI) mouse model, characterized by behavioral abnormalities similar to those in schizophrenia, and potential interactions among HINT1, N-methyl-D-aspartate receptor (NMDAR), and DA type 2 receptor (D2R) may underlie the schizophrenia-like behavioral deficits induced by SI [39, 40].

### 3.2. Inherited Peripheral Neuropathies (IPNs)

IPNs, which affect the peripheral nervous system (PNS), are neuromuscular and neurodegenerative disorders characterized by disrupted communication between the CNS and body. As one of the most common inherited neuromuscular disorders, the prevalence of IPNs is approximately 1 in 2500 [41]. IPNs include a large group of disorders involving multiple genes and complex phenotypes, so the correct diagnosis of each genetic subtype is a thorny problem for clinicians. At present, more than 100 different subtypes of IPNs have been identified, each with its own specific clinical features, pathophysiology, and prognosis. The unidentified mutations make it difficult to apply molecular diagnosis, and therefore, clinical features and developmental patterns are currently used to direct identification of genetic subtypes in patients with IPNs.

One study showed that mutations of HINT1 may be a cause of distal hereditary motor neuropathies [42]. In addition, Zimoń et al. [43] identified eight different mutations of the HINT1 gene in a cohort of 50 autosomal recessive axonal neuromyotonia (ARAN) patients with neuromyotonia (NM) from 33 unrelated nuclear families. NM is characterized by delayed muscular relaxation after voluntary contractions, induced by overexcited motor axons in the PNS [44]. In order to analyze the association between HINT1 and ARAN patients with NM, Zimoń and colleagues [43] screened patients and found a mutation rate at 11% in irrelevant patients with autosomal recessive peripheral neuropathy, which was 76% in ARAN patients with NM. Thus, there is a robust causal genetic association between HINT1 and ARAN patients with NM. However, Horga et al. did not detect variation of the HINT1 gene by direct sequencing of 152 patients with IPNs in England and Spain, indicating a regional specificity in this association [45–47].

Zimoń and colleagues also evaluated the expression levels of HINT1 in mouse tissues, such as heart, lung, and liver [43]. The results showed that HINT1 was enriched in the sciatic nerve in mice, indicating that HINT1 is a vital component of the function of PNS. Furthermore, they implemented *in vivo* genetic complementation analysis by using HINT1 deficit yeast strain (BY8-5c from *Saccharomyces cerevisiae* strain) and then analyzed HINT1 expression levels in lymphoblastoid cell cultures from affected individuals and irrelevant controls, respectively [43], identifying that mutations of HINT1 belong to loss-of-function mutations. Thus, a new genetic subtype was defined based on this functional mutation, namely, autosomal recessive axonal neuropathy with neuromyotonia (ARAN-NM) [43]. Even so, by using knockout mice, Seburn and colleagues demonstrated that HINT1 knockout mice may be useful for studying the biochemical activities of HINT1, but these mice do not provide a disease model or a means for investigating the basis of HINT1-associated neuropathy and neuromyotonia [48]. Therefore, further investigation is needed to determine whether HINT1 functions are species-specific.

### 3.3. Mood Disorders

Mood disorder is featured by obvious and sustained episodes of mania or depression with the clinical manifestations of major depressive disorder (MDD) and bipolar disorder (BP) [49].

Elashoff et al. [50] performed a meta-analysis of 12 microarray studies and concluded that expression of HINT1 was decreased in postmortem brains of patients with BP. A study using HINT1 KO mice demonstrated that KO mice showed decreased depression-like behavior and enhanced cognitive ability. Additionally, KO mice showed abnormalities in the tail suspension test (TST), which could be alleviated by acute administration of the mood-stabilizer valproic acid (VPA) [51]. Increased corticosterone secretion in HINT1 KO mice was also observed [51]. These behavioral and endocrine changes indicate that HINT1 participates in emotional regulation in the CNS, and its absence may lead to manic-like behavior. Furthermore, another study using HINT1 KO mice suggested HINT1 KO mice exhibited behavioral and molecular alterations paralleling those described in BP patients. Thus, HINT1 KO mice could be used as an appropriate model for studying BP and may help identify novel targets and drugs to treat this mental disorder [52].

Interestingly, Martins-de-Souza et al. [53] screened differential protein expressions in the DLPFC of postmortem brains from 24 patients with MDD and 12 controls and detected increased expression of HINT1 in patients with MDD without psychotic symptoms. Moreover, in a study using the chronic mild stress (CMS) depression model to explore the antidepressant effect of oleamide, proteomics analysis showed that the expression level of HINT1 protein in the hippocampus of the CMS group was increased [54]. These results indicate that in different episodes of mood disorders, HINT1 works exactly the opposite.

### 3.4. Anxiety Disorder

There is currently a shortage of clinical studies on the association between HINT1 and anxiety disorder, and results from preclinical studies are not consistent. Barbier et al. [36] conjectured that anxiolytic-like behaviors were included in HINT1 deficiency-induced emotional alterations [51]. While Varadarajulu et al. studied the behaviors of male HINT1 KO mice in a battery of tests. They concluded that *HINT1* KO mice exhibited increased anxiety-like behavior compared with that in WT mice [55]. What is more, Jackson et al. [56] found that in male HINT1 KO mice, the acute administration of nicotine resulted in production of anxiety-like responses rather than its anxiolytic effects, and administration of diazepam failed to induce anxiolytic responses. However, the anxiety-like behaviors described above were not observed in female *HINT1* KO mice, further supporting the aforementioned existence of gender differences in the behavioral impact of HINT1. All results from the anxiety studies were controversial, probably because of deviations in methods, experimental equipment, and animal age (e.g., Wang et al. often use older animals than Varadarajulu et al.).

### 3.5. Pain and Analgesia

The human *μ*-opioid receptor (MOR), a G protein-coupled receptor (GPCR), is the molecular target of morphine-induced analgesia and opiate-related addiction. Guang et al. [57] first discovered the specific interaction between HINT1 and the C-terminus of human MOR using a yeast two-hybrid system. This interaction reduced the desensitization and phosphorylation of MOR. Meanwhile, increased basic pain threshold and enhanced morphine-induced analgesic effects were found in *HINT1* KO mice. However, the dose-response curve indicated that KO mice exhibited a greater extent of tolerance to morphine-induced analgesia than WT mice. In addition, our group and Garzon's research team revealed that HINT1 deficiency could induce abnormalities in the hot-plate test, formalin-induced inflammatory pain, and CCI-induced neuropathic nociception [58–60]. In particular, Garzon and colleagues demonstrated that the inhibitor of HINT1 enzymatic activity, guanosine-5′-tryptamine carbamate (TpGc), significantly enhanced morphine antinociception and alleviated mechanical allodynia but prevented the development of tolerance to opioids [61]. These results show the negative regulatory effect of HINT1 in MOR-mediated morphine-induced analgesia. However, an association study of 2294 patients with cancer pain did not find a correlation between SNP mutations in the *HINT1* gene and opioid dose [62].

### 3.6. Drug Addiction

Association analysis from two independent samples indicates that mutations in the *HINT1* gene are associated with phenotypes of nicotine dependence. Further analysis of mRNA expression in human postmortem brain showed that smoking status and phenotype were associated with HINT1 expression [63]. Chronic nicotine administration elevated HINT1 expression in mouse NAc, which could then be reversed by a nicotine antagonist, mecamylamine, after 24 hours or drug withdrawal after 72 hours [63]. These results show a genetic association between HINT1 and nicotine dependence. Jackson et al. [64] employed the conditioned place preference (CPP) reward test and conditioned place aversion (CPA) test to evaluate emotional and somatic symptoms after nicotine withdrawal. Significant CPA after withdrawal was found in both *HINT1* KO and WT mice. In *HINT1* KO mice, however, nicotine failed to induce significant CPP and somatic withdrawal symptoms (e.g., hyperalgesia) were alleviated. This study could further support the conclusion that HINT1 plays a role in regulating behaviors associated with nicotine reward and withdrawal. However, in an open-label randomized trial of nicotine replacement therapy (NRT) covering 374 nicotine-dependent smokers, the results do not support the relationship between *HINT1* gene mutation and smoking cessation [65].

Relatively few studies have examined the role of HINT1 in addiction induced by other abused drugs. Romanova et al. [66] found that after a single injection of cocaine, HINT1 peak intensities increased significantly in the medial prefrontal cortex (mPFC) of low cocaine responder (LCRs) rats in the open field test. Previous studies showed that the LCRs were more sensitive to cocaine-induced behavioral sensitization compared to high cocaine responders (HCRs) [67, 68]. Increased cocaine CPP [69] and self-administration motivation [70] exhibited by LCRs suggests that LCRs are sensitive to cocaine addiction. Thus, HINT1 is highly expressed in the susceptible phenotype of cocaine addiction. Our recent study has demonstrated that the HINT1 protein, particularly in the NAc, also plays a vital role in methamphetamine-induced CPP [71].

### 3.7. Down's Syndrome (DS)

Weitzdoerfer et al. [72] used two-dimensional gel electrophoresis and mass spectrometry to analyze proteins in cortical tissue from aborted human fetus. They found that different kinds of early life proteins, including HINT1, that participate in neural differentiation, neural migration, and synaptic transmission were deficient in DS.

### 3.8. Brain Aging

Brain aging is one of the major high risk factors for many neurodegenerative disorders such as Alzheimer's disease (AD). Nevertheless, the molecular mechanisms of brain aging are complicated and still unclear. Rassoul et al. [73] analyzed differential transcriptome expression in the temporal cortex of the primate *Microcebus murinus*. Of 695 different genes identified among young healthy animals, old healthy animals, and AD-like animals, approximately 1/3 showed the same expression changes in healthy aging animals and AD-like animals, including the downregulation of HINT1 and HINT2. These findings indicate the possible contribution of HINT1 in the biological process of brain aging.

## 4. Potential Role of HINT1 in Neuroplasticity

As reviewed thus far, HINT1 is implicated in diverse neurological and neuropsychiatric diseases. Related to the latter, our studies have revealed that HINT1 is involved in SI mice model, which could induce behavioral abnormalities related to the core symptoms of certain neuropsychiatric disorders [39, 40]. Neuropsychiatric disorders are a class of diseases closely related to the environment and genetics. One of the core problems in neuropsychiatric disorders is abnormal changes in neuroplasticity [74]. Therefore, it could be hypothesized that HINT1 may play an important role related to neuroplasticity in neuropsychiatric disorders. Thus, HINT1 is a potential promising neuroplasticity mediator in neuropsychiatric diseases.

**Figure 3:**
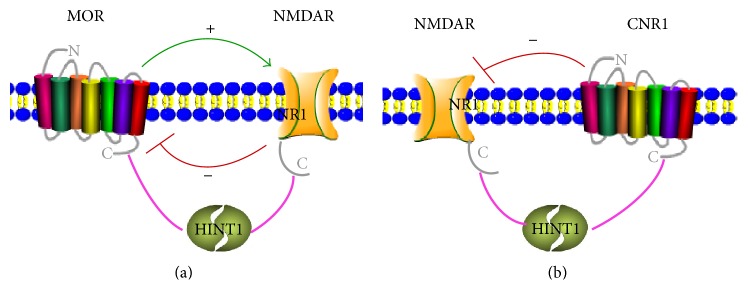
The pattern of HINT1 interacting with GPCRs. (a) HINT1 interacts with the C-terminus of *μ*-opioid receptor (MOR). HINT1 also interacts with the NR1 subunit of NMDAR. To prevent opioids from producing an excessive reduction of neuronal excitability, NMDARs are recruited to the MOR environment, where they become activated to restrain opioid signaling. In this context, HINT1 stabilizes the functional interaction between MOR and NMDAR. (b) HINT1 may also associate with cannabinoid receptor type I (CNR1). CNR1 can negatively regulate NMDAR function when the receptor is coupled to HINT1.

Actually, on one hand, HINT1 could trigger apoptosis independent of its enzymatic activity [14], while there is little research on the exact role of HINT1 in apoptosis. On the other hand, a growing body of evidence suggests that HINT1 acts as a molecular switch regulating the interaction and functional association between GPCRs and NMDARs. For example, HINT1 could stabilize the interaction between MOR/cannabinoid receptor type 1 (CNR1) and NMDARs, promoting (e.g., MOR) or reducing (e.g., CNR1) its glutamatergic activity (Figure 3) [57, 59, 60, 75–83]. HINT1 protein may also participate in conveying information mediated by GPCRs to different signaling pathways, especially the glutamate NMDAR-mediated neurotransmission and functional neural plasticity, such as long-term potentiation (LTP) [60, 76, 84]. Moreover, our accepted study indicated that under both basal and chronic immobilization stress conditions, compared to WT mice, HINT1 KO mice expressed more hippocampal BDNF [85], which is also a key molecule engaged in neuroplasticity [86, 87]. However, to understand the specific role of HINT1 in neuroplasticity, more in-depth study is needed.

## 5. Summary and Prospect

Since HINT1 was discovered to be involved in a variety of biological phenomena, the research interest in this protein has been increasing. Though many studies have aimed to elucidate its roles in cell physiology, the complete range of functions of HINT1 is yet to be determined. The known functions of HINT1, such as tumor suppression, nucleoside transferase, and hydrolase functions, are only a tiny fraction of the whole picture. Currently, treatments for human neuropsychiatric diseases rely on a very limited selection of drugs and therapies, primarily because of our superficial knowledge of the pathogenesis of these diseases. Reviewing the available literature on HINT1, we found that HINT1 is highly related to many neuropsychiatric diseases including schizophrenia, mood disorder, drug addiction, and so on, and HINT1 may participate in neuropsychiatric diseases as a potential neuroplastic mediator. While many studies describe the correlation between HINT1 and neuropsychiatric diseases, few of them describe specific mechanisms. Thus, further study of HINT1 would be of potential value for expanding basic research, diagnosis, and treatment of neuropsychiatric and even psychosomatic diseases.
